# Single Nucleotide Polymorphisms in *Apolipoprotein B, Apolipoprotein E*, and *Methylenetetrahydrofolate Reductase* Are Associated With Serum Lipid Levels in Northern Chilean Subjects. A Pilot Study

**DOI:** 10.3389/fgene.2021.640956

**Published:** 2021-09-20

**Authors:** Anita S. Gálvez, Hugo Ramírez, Pablo Placencia, Claudio Rojas, Ximena Urzúa, Alexis M. Kalergis, Luis A. Salazar, Jorge Escobar-Vera

**Affiliations:** ^1^Laboratorio de Genética, Departamento Biomédico, Facultad de Ciencias de la Salud, Universidad de Antofagasta, Antofagasta, Chile; ^2^Departamento de Genética Molecular y Microbiología, Facultad de Ciencias Biológicas, Millennium Institute on Immunology and Immunotherapy (MIII), Pontificia Universidad Católica de Chile, Santiago, Chile; ^3^Departamento de Endocrinología, Facultad de Medicina, Pontificia Universidad Católica de Chile, Santiago, Chile; ^4^Departamento de Ciencias Básicas, Facultad de Medicina, Centro de Biología Molecular & Farmacogenética, Universidad de La Frontera, Temuco, Chile

**Keywords:** Lipid levels, genetic variants, Apo B, Apo E, MTHFR, polymorphism

## Abstract

Characterization of allelic variants is relevant to demonstrate associations among genetic background and susceptibility to develop cardiovascular diseases, which are the main cause of death in Chile. Association of APOB, APOE, and MTHFR polymorphisms with higher lipid levels and the risk of developing hypertension and cardiovascular diseases have been described. Thus, the aim of this study was to assess genotype distribution and relative allelic frequency of ApoB rs693, ApoE rs7412, ApoE rs429358, MTHFR rs1801131, and MTHFR rs1801133 allelic variants and their effects on lipid profile in young healthy men and women from Northern Chile. A group of 193 healthy subjects were enrolled for this study. Genotyping of rs693 (*APOB*), rs7412 and rs429358 (*APOE*), and rs1801131 and rs1801133 (*MTHFR*) polymorphisms were performed by real time PCR. In addition, lipid profiles were determined and associated to genetic data. The genotype distribution was *APOB* rs693 (CC = 37%, CT = 41%, and TT = 22%), *APOE* rs7412/rs429358 (E4 = 0.06, E3 = 0.91, and E2 = 0.03), *MTHFR* rs1801131 (AA = 57%, AC = 30%, and CC = 13%), and *MTHFR* rs1801133 (CC = 20%, CT = 47%, and TT = 33%). The association of the genetic variants with plasma lipid levels showed that women, but not men, carrying *APOB* mutated allele (T) and Apo E4 allele presented lower values of total cholesterol when compared with C/C homozygous genotype or E3 allele, respectively (*p* < 0.05). In addition, a subgroup analysis revealed that ApoB C/C homozygous women exhibited higher values of HDL-C when compared with men carrying identical genotype (*p* < 0.01). On the other hand, women carrying E4 allele exhibited lower values of triglycerides when compared with male carrying identical genotype (*p* < 0.05). Finally, women carrying mutate allele (C) for *MTHFR* rs1801131 showed lower levels of triglycerides when compared with A/A homozygous genotype (*p* < 0.05) and lower levels of LDL-C for *MTHFR* rs1801133 in females carrying (T) allele when compared with males carrying identical genotype (*p* < 0.05). In summary, the present data showed that *APOB, APOE*, *and* MTHFR single nucleotide polymorphisms are associated to lipid levels in a gender-dependent manner among healthy subjects from Northern Chile, especially in women.

## Introduction

Studies based on genetic markers are frequently used to evaluate the relationships between the presence of an allelic variant and the susceptibility to develop chronic diseases. However, the precise magnitude of inheritance is modified depending on the polygenic model, including other factors such as disease type and age onset (Smolková et al., 2015); therefore, one of the main goals of biomedical research is to correlate genotype with biochemical or molecular abnormalities (Liggett et al., 2007). On this issue, lipoproteins, playing a main function in lipid transport and metabolism (Defesche et al., 2017), along with hyperlipidemia are considerably important in the development of cardiovascular diseases (CVDs) (Homsma et al., 2008), which are the main cause of death in Chile during the past years (DEIS, 2021). In this respect, the National Health Survey showed that around 40% of individuals have a high prevalence of two or more major risk factors, including low high-density lipoprotein cholesterol (HDL-C) concentrations (39%) and hypercholesterolemia (35%), among others (ENS, 2003). Although CVD remains the leading cause of death in both men and women in Chile, the latest report revealed that CVD-related mortality is higher in women (28.9%) than in men (26.2%) (Varleta et al., 2020). In addition, in-hospital mortality due to acute myocardial infarction was significantly greater in women than in men at any age (Nazzal and Alonso, 2013); however, according to some reports, women are not fully aware of this situation (Varleta et al., 2020). Therefore, a better knowledge about lipoprotein allelic variants and their effects to regulate metabolism and transport decreasing cholesterol and lipid blood levels in young healthy individuals, especially in women, may result in improving therapeutic treatments along with preventive public health strategies.

Despite the fact that a genome-wide association study focuses on association of numerous single nucleotide polymorphisms (SNPs) as molecular markers of a disease, analyses of specific genetic variants are helpful in the early detection of individuals carrying genetic susceptibility for hyperlipidemia (Defesche et al., 2017). Apolipoprotein (Apo) B gene, located on the short arm of chromosome 2 with a span of 43 kilobases and 29 exons (Innerarity et al., 1990), is the most important of the atherogenic lipoproteins, having a critical function in the dynamic equilibrium of cholesterol being relevant for the assembly and secretion of very low-density lipoproteins (VLDL) from the liver and uptake of low-density lipoproteins (LDL) mediated by LDL receptor (LDLR). Therefore, elevated Apo B and LDL-cholesterol (LDL-C) levels are risk factors for atherosclerosis; in contrast, low levels of Apo B may contribute to protect against atherosclerosis (Benn, 2009). Specifically, Apo B rs693 genetic variant (*Xba*I, Thr2515Thr) consisted of a change from cytocine (C) to thymine (T) in exon 26, codon 2488 creating a synonymous mutation from ACC to ACT with no change of amino acid residue Thr (Niu et al., 2017). On the other hand, Apo E gene, located on chromosome 19, contains four exons encoding a 34,200-Da protein of 299 amino acids. Apo E is a multifunctional protein synthesized mainly in the liver, taking part in the reverse cholesterol transport and triglyceride metabolism. Apo E binds to hepatic Apo E or LDLR to mediate chylomicrons and VLDL clearances from the plasma (Eichner et al., 2002). Two single-nucleotide polymorphism variants in the Apo E gene (rs429358 and rs7412) will generate different protein isoforms with opposite effects on lipid metabolism because they differ in their receptor-binding activity. Distinction based on cysteine (Cys) or arginine (Arg) at positions 112 and 158 amino acid residue determines three major isoforms commonly referred to as E2 (Cys112/Cys158), E3 (Cys112/Arg158), and E4 (Arg112/Arg158). These isoforms produce six genotypes, including three homozygous (E2/E2, E3/E3 and E4/E4) and three heterozygous (E3/E2, E4/E3 and E4/E2), which produce three isoforms of Apo E, referred to as Apo E2, Apo E3, and Apo E4 (Eichner et al., 2002; Khan et al., 2013). In addition, the enzyme methylenetetrahydrofolate reductase (MTHFR), which catalyzes the conversion of 5,10-methylenetetrahydrofolate to 5-methyltetrahydrofolate, presents several allelic variants associated to modifications in its activity, resulting in hyperhomocysteinemia as an emerging risk factor for various cardiovascular, cerebrovascular, and neurological diseases along with several cancers (Bailey and Gregory, 1999; Liu et al., 2020). MTHFR gene, located in chromosome 1, encoded two enzyme isoforms of 70 and 77 kDa due to multiple transcription start sites (Tran et al., 2002). The two most common MTHFR genetic variants are rs1801133, C677T resulting in alanine (C) to valine (T) substitution with a reduction of enzymatic activity and rs1801131, A1298C resulting in glutamic acid (A) to alanine (C) substitution, showing a significant enzymatic activity decrease in individuals carrying C/C genotype (Abu-Hassan et al., 2019; Mahesh et al., 2019). *MTHFR* rs1801133 polymorphism was associated to elevated risk of myocardial infarction in young and middle-aged Caucasian individuals (Liew and Gupta, 2015); however, this issue remains controversial showing both association between MTHFR rs1801133 and hypertension and the lack of association (Amrani-Midoun et al., 2016; Ghogomu et al., 2016).

Consequently, the current study aims to determine the frequencies of rs693 (*APOB)*, rs7412 and rs429358 (*APOE)*, and rs1801131 and rs1801133 (*MTHFR*) genetic polymorphisms, commonly associated to cardiovascular risk, in healthy men and women from Northern Chile and their possible relationships with plasma lipid levels. Previous studies conducted in our country have investigated some of these genetic polymorphisms in subjects from the Central and Southern regions of Chile. To our knowledge, this is the first study in healthy subjects from Northern Chile.

## Materials and Methods

### Subjects

A group of 193 unrelated subjects (138 females and 55 males) volunteered to be enrolled in the current study. All participants were university students born in the region of Antofagasta (Northern Chile), having good health condition and no diagnosis of CVD, and were informed regarding the study design and goals. All the participants signed a written informed consent before enrolling in the study and blood sample extraction.

Individuals participating in the study required to answer a standardized questionnaire regarding basic health information; the collected data included age, gender, and body weight besides smoking, drinking, recreational drug consumption, exercise habits, and history of CVD based on self-reports. The type of recreational drugs used was not specified. They were all required to have normal fasting glucose levels (lower than 100 mg/dl), systolic blood pressure (SBP) lower than 120 mm Hg, diastolic blood pressure (DBP) lower than 80 mm Hg, and body mass index (BMI) lower than 25. Individuals taking anti-hypercholesterolemic, anti-hypertensive, or hypoglycemic medicines or those who declared to have any personal or family history of CVD were excluded from the study.

Anthropometric and clinical parameters as well as blood sample analyses were processed following standard procedures. Briefly, BMI was defined as the body weight in kilograms divided by the square of the body height in meters and expressed in units of kilograms per square meter. Blood samples were withdrawn and divided in two fractions; a plasma fraction was separated by centrifugation for biochemical analyses and stored at −20°C, and another fraction for analyses of total genomic DNA was conserved at 4°C until processing.

This study has been approved by the Ethics Committee of Universidad de Antofagasta (Chile). Every subject agreed to voluntarily take part in the study by signing a written informed consent. The investigation was performed accordingly to the ethical principles of the World Medical Association Inc (2008).

### Biochemical Measurements

Venous blood samples were withdrawn from antecubital vein after overnight fasting. The plasma levels of glucose, total cholesterol, triglyceride, and HDL-C were quantitated using enzymatic-colorimetric kits commercially available from Human Diagnostics Worldwide, Germany. LDL-C value was calculated using Friedewald’s formula if triglyceride levels did not exceed 400 mg/dl.

### DNA Genotyping

Genomic DNA samples were extracted from peripheral blood leukocytes by using salting out method depicted by Salazar et al. (2001). Genotyping analysis for ApoB, Apo E, and MTHFR polymorphisms was performed by real-time PCR using 4351379 TaqMan SNP Genotyping Assays, Human, SM (ID assays were C_7615420_20 for rs693, C_904973_10 for rs7412, C_3084793_20 for rs429358, C_850486_20 for rs1801131, and C_1202883_20 for rs1801133; Applied Biosystems; Foster City, CA, United States). The PCR reactions were carried out accordingly to standard conditions provided by the manufacturer. In summary, assays contained 1 μl DNA (20 ng), 6.25 μl 2 × Taqman PCR master mix, 0.625 μl 20 Taqman genotyping assay, and 5.125 μl nuclease-free water for 13 μl total volume. Thermal cycling conditions for real-time system were initial denaturation at 95°C for 10 min, followed by 50 cycles of denaturation at 95°C for 15 s, annealing and extension at 60°C for 1 min, and a final extension at 60°C for 30 s.

For analytical purposes, the six APOE genotype groups (E2/E2, E3/E2, E3/E3, E4/E3, E4/E4, and E4/E2) were classified into three groups. The E3/E3 genotype occurs at high frequency in the population and it is considered the normal variant (Liehn et al., 2018). The E2/E2 and E3/E2 genotypes were combined and presented as E2 carriers. Since E4/E4 genotype individuals were absent in the study, the E4/E3 genotype is presented as E4 carriers (Wu et al., 2007). Previous studies have shown that the impact of the E2 allele on serum lipids is greater than that of the E4 allele (Wilson et al., 1994); therefore, individuals carrying E4/E2 genotype were excluded from the analysis.

### Statistical Analyses

Genotype and allele frequencies were acquired by direct gene counting. The genotype distribution for Hardy–Weinberg equilibrium (HWE) was evaluated by χ^2^ analysis. The Shapiro–Wilk test was performed to assess for normal distribution of continuous variables. Data were presented as mean ± SD. Comparison of continuous variables was accomplished using Student’s *t*-test or ANOVA one-way test. The multiple comparisons were accomplished by the Bonferroni method. The Sigma Stat statistical software (Systat Software Inc., San Rafael, CA, United States) was used for statistical analyses. A p value < 0.05 was considered as statistically significant.

## Results

Anthropometric features and clinical parameters of 193 subjects (28% male and 72% female) enlisted in the study are summarized in Table 1. The average age was 22.5 ± 8.8 years (22.2 ± 7.6 and 22.6 ± 9.2 years for male and female, respectively). Every volunteer had normal value for anthropometric BMI 23.0 ± 2.5 kg/m^2^ (23.2 ± 2.2 kg/m^2^ and 22.4 ± 2.2 kg/m^2^ for male and female, respectively). Clinical parameters were normal, showing SBP of 116.2 ± 14.6 mm Hg (118.7 ± 12.9 mm Hg and 115.4 ± 15.2 mm Hg for male and female, respectively) and DBP of 70.1 ± 11.2 mm Hg (71.2 ± 13.3 mm Hg and 69.9 ± 10.3 mm Hg for male and female, respectively). Plasma glucose and lipid levels were all within the reference range (measured as mg/dl) (glucose 86.1 ± 28.0; total cholesterol 139.7 ± 37.4; triglycerides 83.7 ± 55.6; HDL-cholesterol 56.4 ± 23.8; LDL-cholesterol 67.7 ± 39.0). Analysis by gender is shown in Table 1. No significant differences were found among groups.

**TABLE 1:** Anthropometric features and clinical parameters of the studied individuals.

**Parameter**	**Total (193)**	**Male (55)**	**Female (138)**
Age (years)	22.5 ± 8.8	22.2 ± 7.6	22.6 ± 9.2
BMI (kg/m^2^)	23.0 ± 2.5	23.2 ± 2.2	22.4 ± 2.2
SBP (mm Hg)	116.2 ± 14.6	118.7 ± 12.9	115.4 ± 15.2
DBP (mm Hg)	70.1 ± 11.2	71.2 ± 13.3	69.9 ± 10.3
Glucose (mg/dl)	86.1 ± 28.0	84.7 ± 23.6	86.7 ± 28.9
Cholesterol (mg/dl)	139.7 ± 37.4	137.1 ± 31.7	141.1 ± 39.9
Triglycerides (mg/dl)	83.7 ± 55.6	77.7 ± 33.8	87.4 ± 65.2
HDL-cholesterol (mg/dl)	56.4 ± 23.8	49.2 ± 11.8	59.2 ± 26.5
LDL-cholesterol (mg/dl)	67.7 ± 39.0	72.4 ± 32.0	65.5 ± 42.2
Cigarette smoking (%)	22	20	24
Alcohol consumption (%)	69	74	66
Drugs consumption (%)	14	12	15
Physical activity (%)	55	67	50

*Number of subjects in parentheses. Values are expressed as mean ± SD. Physical activity performed at least once a week. BMI, body mass index; SBP, systolic blood pressure; DBP, diastolic blood pressure; HDL-C, high-density lipoprotein cholesterol; LDL, low-density lipoprotein cholesterol.*

Genotype and allele frequencies for the studied polymorphisms are shown in Table 2. The relative allele frequency for *APOB* rs693 polymorphism was 0.42 for mutated allele (T). The relative allele frequencies for APOE rs7412/rs429358 polymorphisms were 0.06, 0.91, and 0.03 for E4, E3, and E2 alleles, respectively. The relative allele frequencies for *MTHFR* rs1801131 and rs1801133 polymorphisms were 0.28 and 0.56 for mutated alleles (C) and (T), respectively. Overall, *MTHFR* rs1801133 was the only genetic variant to show the distribution pattern predicted by HWE (*p* = 0.6), whereas the other three genotypes differed from the Hardy–Weinberg prediction (*p* < 0.05).

**TABLE 2:** Genotype distribution and relative allele frequency for ApoB, ApoE, and MTHFR polymorphisms.

	**Genotype frequency (%)**				**P-value**	**Allele frequency**	
		**CC**	**CT**	**TT**		**C**	**T**
ApoB (rs693) *n* = 189	Total	70 (37)	78 (41)	41 (22)	0.03	0.58	0.42
	Male	18 (37)	24 (49)	7 (14)			
	Female	52 (37)	54 (39)	34 (24)			

	**Genotype frequency (%)**						**P-value**	**Allele frequency**		
		**E4/3**	**E4/2**	**E3/3**	**E3/2**	**E2/2**		**E4**	**E3**	**E2**

ApoE (rs7412, rs429358) *n* = 127	Total	13 (10.2)	1 (0.8)	107 (84)	5 (3.9)	1 (0.8)	<0.05	0.06	0.91	0.03
	Male	6 (17.6)	1 (2.9)	27 (79.5)	0	0				
	Female	7 (7.5)	0	80 (86)	5 (5.4)	1 (1.1)				

	**Genotype frequency (%)**				**P-value**	**Allele frequency**	
		**AA**	**AC**	**CC**		**A**	**C**

MTHFR (rs1801131) *n* = 186	Total	106 (57)	56 (30)	24 (13)	0.001	0.72	0.28
	Male	29 (60)	12 (25)	7 (15)			
	Female	77 (56)	44 (32)	17 (12)			

	**Genotype frequency (%)**				**P-value**	**Allele frequency**	
		**CC**	**CT**	**TT**		**C**	**T**

MTHFR (rs1801133) *n* = 193	Total	39 (20)	91 (47)	63 (33)	0.6	0.44	0.56
	Male	12 (22)	28 (51)	15 (27)			
	Female	27 (20)	63 (46)	48 (35)			

*χ^2^ analysis was used to test Hardy–Weinberg equilibrium. Genotype E4/E4 was not found and genotype E4/E2 was excluded from the study; therefore, the E4/E3 is presented as E4 carriers. The E3/E2 and E2/E2 genotypes were combined and presented as E2 carriers.*

Based on previous reports (Utermann, 1987; Lagos et al., 2015; Han et al., 2016), we studied the potential association of ApoB rs693, ApoE rs7412, Apo E rs429358, MTHFR rs1801131, and MTHFR rs1801133 allelic variants with plasma lipid profile. No changes in lipid levels were observed for the entire group of subjects studied. The results are shown in Table 3. However, when the analysis was performed by gender, the female group carrying ApoB C/T heterozygous genotype exhibited significant lower levels of total cholesterol (*p* < 0.05) compared with those female carrying C/C genotype. In addition, we observed higher levels of HDL-C in females carrying C/C homozygous genotype compared with males carrying identical genotype (*p* < 0.01). On the other hand, analysis of ApoE rs7412 and ApoE rs429358 genetic variants exhibited significant lower levels of total cholesterol along with LDL-C in females carrying E4 compared with E3 allele (*p* < 0.05). Higher levels of triglycerides were found in males carrying E4 compared with E3 allele (*p* < 0.01); on the contrary, higher level of triglycerides, although not significant, were observed in females carrying E2 allele. In addition, lower levels of triglycerides were found in females carrying E4 allele compared with males carrying identical genotype. Analysis of *MTHFR* rs1801131 genetic variant showed significant lower value of triglycerides in females carrying A/C heterozygous compared with A/A homozygous genotype (*p* < 0.05). On the other hand, females carrying MTHFR rs1801133 T/T homozygous genotype showed significant lower values of LDL-C compared with males carrying identical genotype (*p* < 0.05). A comparative analysis of allele frequencies of the four genetic variants found in the population worldwide along with people living in different regions of Chile is shown in Table 4.

**TABLE 3:** Lipid profile for ApoB, ApoE, and MTHFR polymorphisms in healthy individuals from Northern Chile.

		**TC**			**TG**			**HDL-C**			**LDL-C**		
		**C/C**	**C/T**	**T/T**	**C/C**	**C/T**	**T/T**	**C/C**	**C/T**	**T/T**	**C/C**	**C/T**	**T/T**
ApoB (rs693)	Total	147.8 ± 39.5	140.5 ± 41.4	138.5 ± 31.4	78.9 ± 30.7	76.2 ± 38.9	80.9 ± 38.4	53.7 ± 15.5	55.2 ± 18.0	52.6 ± 14.6	77.4 ± 40.2	67.2 ± 34.4	69.7 ± 31.6
	Male	139.2 ± 27.9	142.5 ± 45.6	130.3 ± 25.5	87.9 ± 37.1	72.7 ± 37.4	79.7 ± 35.9	45.5 ± 11.7	52.8 ± 11.8	47.1 ± 11.9	74.9 ± 32.8	70.0 ± 28.9	67.3 ± 22.2
	Female	151.9 ± 43.9	135.6 ± 36.9*	140.9 ± 32.9	78.4 ± 41.4	75.3 ± 38.5	77.9 ± 36.8	57.2 ± 14.9^ǂǂ^	56.7 ± 19.9	53.9 ± 15.2	73.0 ± 31.3	66.8 ± 38.5	71.5 ± 33.9

		**E4**	**E3**	**E2**	**E4**	**E3**	**E2**	**E4**	**E3**	**E2**	**E4**	**E3**	**E2**

ApoE (rs7412, rs429358)	Total	124.2 ± 35.2	134.8 ± 29.4	137.8 ± 18.4	86.7 ± 46.4	74.9 ± 35.5	82.8 ± 30.8	53.2 ± 13.6	58.6 ± 42.7	44.7 ± 13.9	61.5 ± 33.4	68.4 ± 33.9	71.9 ± 18.4
	Male	142.8 ± 31.1	134.5 ± 33.3	N.D.	114.1 ± 14.2**	68.2 ± 30.1	N.D.	45.8 ± 8.4	48.2 ± 13.6	N.D.	74.2 ± 29.9	72.6 ± 32.2	N.D.
	Female	107.6 ± 28.3*	135.1 ± 28.9	137.8 ± 18.4	70.4 ± 55.9^ǂ^	74.6 ± 29.5	82.8 ± 30.8	60.7 ± 15.5	57.0 ± 17.6	44.6 ± 13.9	48.9 ± 34.4*	73.1 ± 64.8	71.9 ± 18.4

		**A/A**	**A/C**	**C/C**	**A/A**	**A/C**	**C/C**	**A/A**	**A/C**	**C/C**	**A/A**	**A/C**	**C/C**

MTHFR (rs1801131)	Total	136.6 ± 37.9	141.9 ± 40.3	138.8 ± 28.7	88.5 ± 62.4	73.3 ± 36.9	86.1 ± 28.9	65.8 ± 38.7	66.9 ± 38.2	55.9 ± 22.3	67.6 ± 40.8	72.2 ± 38.3	62.3 ± 27.6
	Male	130.9 ± 33.2	143.6 ± 33.5	136.2 ± 29.7	76.7 ± 39.5	83.5 ± 27.6	96.7 ± 18.6	67.9 ± 44.8	63.4 ± 31.2	48.9 ± 15.1	71.8 ± 39.3	77.1 ± 30.3	70.3 ± 31.1
	Female	138.7 ± 39.6	141.4 ± 42.4	140.0 ± 29.2	92.8 ± 68.7	70.3 ± 39.0*	81.2 ± 32.1	66.8 ± 35.7	67.9 ± 40.3	62.5 ± 19.3	67.9 ± 40.5	70.9 ± 40.5	63.3 ± 22.1

		**C/C**	**C/T**	**T/T**	**C/C**	**C/T**	**T/T**	**C/C**	**C/T**	**T/T**	**C/C**	**C/T**	**T/T**

MTHFR (rs1801133)	Total	146.7 ± 30.7	136.7 ± 42.3	138.2 ± 34.0	92.6 ± 70.9	77.4 ± 49.6	88.1 ± 45.7	65.2 ± 31.3	63.1 ± 35.9	62.3 ± 36.2	72.2 ± 31.7	67.4 ± 46.1	65.2 ± 39.5
	Male	134.6 ± 28.4	127.7 ± 31.6	146.9 ± 39.8	74.7 ± 42.4	77.9 ± 32.7	96.5 ± 30.3	57.7 ± 30.9	57.4 ± 40.4	63.8 ± 39.9	74.4 ± 31.6	61.3 ± 39.8	85.3 ± 36.9
	Female	151.4 ± 31.1	140.9 ± 46.0	136.4 ± 32.2	99.7 ± 73.8	77.8 ± 55.7	85.4 ± 49.5	69.2 ± 31.2	67.5 ± 35.5	61.9 ± 35.5	71.9 ± 32.7	70.2 ± 48.5	59.5 ± 38.6^ǂ^

Value are expressed in mg/dl and represented as mean ± SD. The E2/E2 and E2/E3 genotypes were combined and presented as E2 carriers. Since E4/E4 individuals were absent, the E3/E4 genotype was presented as E4 carriers. The E2/E4 genotype was excluded from the analysis. Data were analyzed by one-way ANOVA, followed by a *post hoc* Bonferroni or Student *t*-test. *p < 0.05 when compared with C/C or A/A alleles; *p < 0.05 or **p < 0.01 when compared with E3 allele; ^ǂ^*p* < 0.05 or ^ǂǂ^*p* < 0.01 when compared with the same genotype by gender. N.D.: not determined.

**TABLE 4:** Allele frequencies for *APOB*, *APOE*, and *MTHFR* polymorphisms in population worldwide and Chilean subjects from different regions.

**Population**	**Apo B rs693**			**Apo E rs7412/rs429358**				**MTHFR rs1301131**			**MTHFR rs1801133**		
	**Sample size**	**C**	**T**	**Sample size**	**E4**	**E3**	**E2**	**Sample size**	**A**	**C**	**Sample size**	**C**	**T**
African	11,472	0.77	0.23					11,466	0.83	0.17	11,720	0.88	0.12
Asian	842	0.94	0.06					884	0.75	0.25	3,984	0.66	0.34
European	207,254	0.51	0.49					211,780	0.69	0.31	322,326	0.65	0.35
Latin American	9,062	0.63	0.37					5,020	0.81	0.19	7,238	0.55	0.45
*Chileans	626	0.62	0.38					626	0.77	0.23			
Northern Chile (Antofagasta)	189	0.58	0.42	127	0.06	0.91	0.03	186	0.72	0.28	193	0.44	0.56
^ǂ^Central Chile				146	0.09	0.88	0.03				146	0.52	0.48
^ǂǂ^Central Chile								105	0.79	0.21			
^ǂǂǂ^Central Chile											184	0.60	0.40
^+^Southern Chile				116	0.18	0.80	0.02						

All data were retrieved from the ALFA project. Release version: 20201027095038, except those indicated. *Eyheramendy et al. (2015). ^ǂ^Roco et al. (2015). ^ǂǂ^Báez et al. (2010). ^ǂǂǂ^Nitsche et al. (2003). ^+^Lagos et al. (2015).

## Discussion

This is the first study highlighting the frequency of ApoB, ApoE, and MTHFR genetic variants and their relationship with plasma lipid levels in young healthy individuals from Antofagasta city (Northern Chile). In the studied group, the relative frequency for mutated allele (T) of rs693 (ApoB) was 0.42, similar to that previously described in Chile (Eyheramendy et al., 2015) and not very different of that found in individuals from Brazil (Nakazone et al., 2009; Tamburus et al., 2018). However, this rate was different from the allele frequency found in Asian individuals as shown in Table 4 and depicted by Niu et al. (2017). For ApoE alleles, the most frequent genotype was E3/3 (84%), followed by E4/3 (10.2%), and E3/2 (3.9%). These data were similar to those described by Roco et al. (2015) in healthy individuals from the central region of Chile; however, we did not detect any subject with genotype E4/4. Similar frequencies were observed in Japanese, Chinese, and Mexican-American populations (Eichner et al., 2002). On the other hand, our data were different from those previously described in Caucasians from Eastern Europe and Amerindian hypercholesterolemic patients from Southern Chile (Eichner et al., 2002; Lagos et al., 2015). The analysis of MTHFR rs1801131 minor allele (C) frequency was 0.28, comparable with those previously described by Báez et al. (2010) and Eyheramendy et al. (2015) in Chile and also similar to those found in American (Mahesh et al., 2019), European, and Asian individuals as shown in Table 4. In addition, MTHFR rs1801133 minor allele (T) frequency was 0.56, different to the one depicted in healthy individuals from the central region of Chile (Nitsche et al., 2003; Roco et al., 2015). Overall, those frequencies are very different from those described by Liew and Gupta (2015) in other regions of the world as shown in Table 4. The differences observed in ApoE and MTHFR rs1801133 allelic frequencies in individuals from the northern region of Chile compared with other regions might be explained based on the genetic background of Chilean people as a consequence of some miscegenation events where larger European and Native-American and smaller African ancestry contributed to admixing, showing that African ancestry contribution is higher in the northern region (3.9%) and diminished in southern regions (1.6%), while European ancestry contribution is higher in the central region and Native-American ancestry prevails in the southern region (Fuentes et al., 2014; Ruiz-Linares et al., 2014; Eyheramendy et al., 2015). Altogether, these observations indicate that the genetic background of Chilean subjects is unevenly spread throughout the country, and this could explain why the studied allelic variants exhibited a geographic dependent pattern as was previously demonstrated for *LDLR* and *PCSK9* polymorphisms (Rojas et al., 2019).

Regarding lipid profiles, no association of any of the genetic variants with total cholesterol, triglycerides, HDL-C, and LDL-C profile was found among the complete studied group of individuals, similar to those previously described (Nakazone et al., 2009; Roco et al., 2015; Mahesh et al., 2019); however, some associations appeared when the analyses were performed for each gender separately. For Apo B genetic variant, C/C genotype was associated with higher total cholesterol along with HDL-C levels in females and therefore may be a risk factor that predisposes to CVD. A similar association was described by Nakazone et al. (2009) in a group of control individuals that consisted of 64% females; however, other authors depicted a similar association in males but not in females (Niu et al., 2017). To this respect, association of ApoB rs693 and lipid levels is controversial since this genetic variant is a silent mutation that cannot directly affect lipid metabolism; however, association with higher levels of total cholesterol, triglyceride, and LDL-C and lower levels of HDL-C has been reported, suggesting that gender, ethnicity, and health status might modulate its association to lipid levels (Peacock et al., 1992; Benn et al., 2008; Niu et al., 2017; Tamburus et al., 2018), but some of those studies presented association of high lipid levels with males, which are opposite to our findings.

In addition, for ApoE genetic variants, E3 allele carriers showed a total cholesterol level similar to that previously described by Roco et al. (2015); however, E4 allele is associated with lower levels of total cholesterol, triglycerides, and LDL-C exclusively in women, whereas lipid level profiles in E2 allele carriers were not different from those observed in E3 allele carriers. These data were not consistent with those found in the general population, suggesting an association of E4 allele with high total cholesterol and LDL-C concentrations (Utermann, 1987) and therefore with high risk of CVD (Eichner et al., 2002; Larifla et al., 2017) or no association at all (Roco et al., 2015). On the other hand, gender-specific studies exhibited high triglyceride levels in men carrying both E2 and E4 alleles (Wilson et al., 1994) similar to our observations for E4 allele, although, as we previously stated, the limitation of a small sample size did not produce any males with E2 allele; therefore, comparisons were done with E4 allele exclusively. On the contrary, Kolovou et al. (2009) depicted increased total cholesterol in both women and men having E4 allele, which is opposite to our findings.

Finally, MTHFR genetic variants showed a gender-specific association with triglyceride (rs1801131) and LDL-C (rs1801133). MTHFR rs1801133 polymorphism has been thoughtfully studied because of its health implications. In general, (T) allele carriers showed higher total cholesterol and LDL-C in a study including Caucasian, Asian, African, and other ethnicities; however, no association with lipid profile was described in Mexican and American individuals carrying (T) allele (Leal-Ugarte et al., 2019; Mahesh et al., 2019), although authors proposed statins’ effect on lowering lipid levels, and therefore no association. Analyses of different subgroups showed significant associations with higher levels of total cholesterol along with LDL-C detected exclusively in females (Luo et al., 2018); however, once again these data are not consistent with our findings showing lower levels of LDL-C in (T) allele carriers. On the other hand, MTHFR rs1801131 showed a significant association with lower levels of HDL-C (Mahesh et al., 2019) and higher levels of total cholesterol and LDL-C in individuals having C/C genotype; in this case, those results were dependent of hypermethylated promoter (Santana Bezerra et al., 2019). However, once again our data were opposite, showing only lower triglyceride levels in females carrying (C) allele.

Overall, these apparent differences in the association of genetic polymorphisms and lipid profile with previous reports may be attributable to the peculiar genetic background admixing existing in the studied individuals. Moreover, the underlying results showed that healthy age-matched subjects from central and southern regions of Chile exhibited lower level of lipids (Arteaga et al., 2010; Caamaño Navarrete et al., 2015), probably due to seasonal variations that produce higher values in winter and lower values in summer (Kreindl et al., 2014; Zhou et al., 2016). Since the northern region of Chile has a hot and dry desertic weather with temperature range 3 to 5°C higher than other regions and no seasons (McKay et al., 2003), it could account for lower lipid values found in our study. Considering these geographical differences on lipid values, the effects of the studied genetic variants on lipid-lowering therapy in subjects with hypercholesterolemia need to be investigated. Several authors have demonstrated the effect of ApoB (Guzmán et al., 2000; Ye et al., 2003), ApoE (Ye et al., 2003; Tavintharan et al., 2007; Lagos et al., 2015), and MTHFR (Maitland-van der Zee et al., 2008; Jiang et al., 2013) polymorphisms on response to statins (HMG-CoA reductase inhibitors), specifically on the pharmacodynamics of these drugs. For example, Ye et al. (2003) observed that the relative frequency of ApoB rs693 genetic variant is high in individuals with hyperlipidemia, in whom the cholesterol-lowering efficacy is diminished after treatment of simvastatin. In relation to APOE variants, Lagos et al. (2015) showed that E4/3 genotype carriers exhibited lower cholesterol reduction compared with E3/3 genotype (LDL-C: −18% vs. −29%, *p* < 0.001), when the hypecholesterolemic subjects were treated with atorvastatin. Finally, in relation to MTHFR gene polymorphisms, Maitland-van der Zee et al. (2008) observed that subjects carrying C/C genotype for MTHFR rs1801133 genetic variant exhibited a significantly protective effect against CHD [0.71 (95% CI 0.58–0.87)] when the patients were treated with pravastatin. Similarly, Jiang et al. (2013) showed that MTHFR rs1801133 genetic variant contributes to the effects of simvastatin in Chinese subjects with primary hyperlipidemia.

In summary, our data showed associations between ApoB (rs693), ApoE (rs7412 and rs429358), and MTHFR (1801131 and 1801133) single nucleotide polymorphisms with lipid levels in healthy individuals from Northern Chile, especially in women. However, to confirm the influence of any of the studied genetic variants on lipid profile, enrolling a greater number of individuals in a case–control study is required. Finally, we propose that understanding new candidate genes that predispose to CVD might allow to elaborate risk score tables to improve therapeutic and preventive strategies. The clinical utility of these genetic polymorphisms in the prediction of cardiovascular risk or the influence on lipid-lowering therapy needs to be investigated in a future study including a larger number of subjects, considering a similar distribution of males and females. Moreover, future directions for this study need to include functional studies.

## Data Availability Statement

The datasets analyzed during the current study are available from the corresponding author on reasonable request.

## Ethics Statement

The studies involving human participants were reviewed and approved by Comité de Ética en Investigación Científica, Universidad de Antofagasta. The patients/participants provided their written informed consent to participate in this study.

## Author Contributions

JE-V conceived and designed the study. HR, CR, PP, and XU performed the experiments. HR, PP, CR, and ASG analyzed the data. LAS participated in the design of the study, contributed reagents/materials, and analysis tools. LAS and AMK reviewed and edited the manuscript. ASG and JE-V wrote the paper. All authors have read the article and agreed with content.

## Conflict of Interest

The authors declare that the research was conducted in the absence of any commercial or financial relationships that could be construed as a potential conflict of interest.

## Publisher’s Note

All claims expressed in this article are solely those of the authors and do not necessarily represent those of their affiliated organizations, or those of the publisher, the editors and the reviewers. Any product that may be evaluated in this article, or claim that may be made by its manufacturer, is not guaranteed or endorsed by the publisher.

## Abbreviations

ANOVA: Analysis of variance
Apo B: Apolipoprotein B
Apo E: Apolipoprotein E
BMI: Body Mass Index
CVD: cardiovascular diseases
DNA: Deoxyribonucleic acid
dNTPs: deoxyribonucleotides
DBP: Diastolic blood pressure
HBP: high blood pressure
HDL-C: high-density lipoprotein-cholesterol
HWE: Hardy Weinberg equilibrium
IAM: acute myocardial infarction
LDL-C: low-density lipoprotein-cholesterol
MTHFR: methylenetetrahydrofolate reductase
MgCl2: Magnesium chloride
PCR: Polymerase chain reaction
SNP: Single nucleotide polymorphism
SBP: Systolic blood pressure
VLDL: very low-density lipoproteins.
